# Effects of Weight Loss on Psoriasis: A Review of Clinical Trials

**DOI:** 10.7759/cureus.3491

**Published:** 2018-10-24

**Authors:** Hammam A Alotaibi

**Affiliations:** 1 Research, Prince Sultan Military Medical City, Riyadh, SAU

**Keywords:** psoriasis, weight loss, exercise, diet, obesity, immunotherapy

## Abstract

Psoriasis is a chronic autoimmune disease that affects the skin and joints. It typically presents as abnormal skin patches characterized by red, scaly, and very itchy spots. It affects patients in different manners with different severities ranging from small spots or spots that cover a larger area of the skin. Due to the negative impact psoriasis has on the quality of life, many patients are exploring other options that can help improve their symptoms. Among those is weight reduction. This review is aimed at providing an overview of the published clinical literature to give physicians an indication of what the answer could be. Moreover, since obesity is correlated with psoriasis vulgaris it is thus also the subject of investigation in this review.

This is a literature review conducted to answer the following question: what are the effects of weight loss on the degree of plaque psoriasis recorded in clinical trials published from 1990 to December 2017. The objective of this study is to find the relationship between the severity of psoriasis and weight.

Ten clinical trials met the inclusion criteria of this review and were included in the final analysis.

Diet and exercise are worthy of consideration as adjunct treatments for psoriasis. Diet and exercise improve the overall health of the patients, are effective in combating oxidative stressors, and also show a positive impact on the Psoriasis Area and Severity Index (PASI) scores of patients with psoriasis.

## Introduction and background

Psoriasis is a chronic autoimmune disease that affects the skin and joints. It typically presents as abnormal skin patches  characterized by red, scaly, and very itchy spots. It affects patients in different manners with different severities ranging  from small spots or spots that cover a larger area of the skin [1]. The majority of cases with psoriasis have the subtype psoriasis vulgaris (scaling psoriasis) and this subtype is one of many subtypes, some of which are pustular, inverse, erythrodermic, and guttate, which accounts for only 10% of the cases [2]. Psoriasis occurs worldwide and affects people irrespective of age [3]; it was even recorded to be present at birth in some cases [4, 5]. The prevalence varies considerably worldwide and among different ethnic and age groups. In the United States, for example, psoriasis affects only 2% of the population, while in Japan it is very low, and it is almost nonexistent in aboriginal Australians and Indians from South America. There is a genetic aspect to the disease where the risk of redeveloping it if one of twins has it is higher than in the general population [6]. Psoriasis has a variable morphology, distribution, severity, and course. It is a papulosquamous disease that can be separated into two categories depending on the scaling size: it is either less than 1 cm or more than 1 cm, and it is defined as scaling papules or plaques, respectively. Lesions of psoriasis are unique among dermatologic conditions in that they are very well demarcated, red, circular with grey or silver dry scale [7]. The body distribution of psoriasis includes the scalp, elbows, knees, lumbosacral area, and in the body folds. The scaling is distributed symmetrically and can develop in sites of injury, which is known as the Koebner phenomenon [8]. Psoriasis has no cure, however, there are several treatments that can affect the course of the disease and improve symptoms, like immune system suppressing medications such as methotrexate or topical steroid creams to improve symptoms. Other treatments used are vitamin D3 cream and ultraviolet light [9]. The disease is associated with an increased risk of developing psoriatic arthritis, cardiovascular disease, Crohn’s disease, and lymphomas. Depression can also be co-morbid with psoriasis especially in teenage females with a visible scaling. The  disease can be worsened with certain conditions such as stress and changing of seasons. Alcohol consumption, hot water, and scratching the psoriatic skin are also factors that worsen the disease [10].

This review aims at studying the effects of weight loss on the symptoms of psoriasis in obese patients.

## Review

This is a literature review designed to answer the question: what are the effects of weight loss on the degree of plaque psoriasis recorded in clinical trials published from 1990 to December 2017. The studies were required to be clinical trials, written in English, and include a sample of adult males and females.

The databases used include PubMed, Medline, and the Cochrane collaboration. The keywords included “psoriasis”, “scaling”, “scales”, “scale”, “plaques”, “plaque”, “vulgaris”, “obesity”, and “weight loss”.

Every study was assessed separately, and the results were organized chronologically. The statistics and methodology of each study were evaluated, and the conclusion was judged based on the quality and reliability of results. The results were not pooled in this review and will be evaluated qualitatively only.

Using Ovid, applying the limitations "published between 1990 and December 2017" and combining the search yielded 14 results. Ten studies were found to be clinical trials that met the inclusion criteria of this review. The discussion of the studies is presented in the following section. Their results are summarized chronologically in Table 1.

In 2014, Debaneh and colleagues conducted a literature review of observational and clinical literature on the effects of  weight loss on the severity of psoriasis. This paper is used as the benchmark for work published prior to 2014. Since then, five articles were published on the same topic, which are the focus of this paper [11].

**Table 1:** Summary of clinical trials BMI: body mass index, BSA: Body surface area, CASPAR: Classification criteria for psoriatic arthritis, CRP: C-reactive protein, DLQI: dermatology life quality index, DMARDS: disease-modifying antirheumatic drugs, ESR: erythrocyte sedimentation rate, Kcal: kilocalorie, LDL: low density lipoprotein, LED: low energy diet, MTX: methotrexate, PASI: psoriasis area and severity index, RCT: randomized clinical trial, SHD: standard hospital diet, TNF: tumor necrosis factor, VAS: visual analogue scale for pain.

Study	Design	Intervention	Size	Inclusion Criteria	Results
Rucevic et al., 2003 [12]	Controlled trial	Dietary intervention: low energy diet (LED): 800 kcal vs. Standard hospital diet (SHD): 2100 kcal	LED: (n=42) SHD: (n=40)	Hospitalized patients. No metabolic or liver disease. Plaque psoriasis for more than 10 years. BSA≥30%	At 4 weeks no weight reduction changes between either group. LED showed significant reduction in LDL which is correlated with improved psoriasis (p<0.05).
Gisondi et al., 2008 [13]	RCT (Blind investigator)	Cyclosporine + Low caloric diet (intervention) VS. Cyclosporine only (control)	Intervention: (n=30) Control: (n=31)	Plaque psoriasis. PASI≥10% BMI≥30 BSA≥10%	At week 24, 7% weight reduction in intervention group, 0.2% in control. PASI 75=66.7% in intervention group vs 29% in control (P<0.001).
Di Minno et al., 2012 [14]	RCT (Blind investigator)	TNF-Alpha inhibitor + either: Low calorie 1500 kcal diet (intervention) Free diet (control)	Intervention: (n=63) Control: (n=63)	Psoriatic arthritis (CASPAR criteria) Failed DMARDS	At 24 weeks, low calorie diets achieved better weight control and better ESR and VAS reduction compared to controls.
Giglio et al., 2012 [15]	RCT (Blinded)	Low calorie diet (1200-1600 kcal) vs. Routine diet. Previous MTX patients.	Intervention: (n=22) Control: (n=20)	Plaque psoriasis BMI≥30	At weeks 12 and 24, intervention group showed more weight reduction than control. No other changes were noted.
Kimball et al., 2012 [16]	RCT (Blind investigator)	Phototherapy 3x/week plus either: Ornish diet South beach diet No specific diet (control)	Ornish: (n=10) South beach: (n=10) Control: (n=10)	Plaque psoriasis BMI≥20 PASI≥10	Dietary intervention at 12 weeks showed significant weight reduction compared to control. No significant change in mean PASI between groups.
Jensen et al., 2013 [17]	RCT	Anti-psoriatic treatment + either: Low calorie diet (800-1000 kcal) Routine diet (control)	Intervention: (n=30) Control: (n=30)	Plaque psoriasis BMI≥27	At 16 weeks, intervention group lost more weight 15.4 kg and achieved better PASI (p=0.06) and DLQI (p=0.02) compared to control.
Roongpisuthipong et al., 2013 [18]	Uncontrolled trial	Low calorie diet with topical steroids	(n=10)	Plaque psoriasis Metabolic syndrome BMI≥30	At 12 weeks, patients lost 9.6% body weight. PASI 50=50%, DLQI=62.5%.
Naldi et al., 2014 [19]	RCT (Blind assessor)	Systemic treatment + either: Dietetic plan - diet and exercise- (intervention) Informative plan - advice about importance of diet-(control)	Intervention (n=151) Control: (n=152)	Plaque psoriasis BMI≥25 No other diagnosis No ongoing weight loss treatment	Change in PASI scores at 20 weeks from baseline showed a change in median PASI of 48% in intervention vs 25.5% in the control group (p=0.02).
Al-Mutairi & Nour, 2014 [20]	RCT	TNF-Alpha inhibitors + either: Low calorie diet: ≤1000 kcal (intervention) or Normal diet (control group)	Intervention (n=131) Control (n=131)	Obese patients Stable plaque psoriasis, PASI 20:50 Receiving biologic therapy (anti TNF-a)	At week 24, the intervention group lost 12.1 kg, achieved 84% improvement in PASI scores compared to 1.5 kg and 69%, respectively, for the control group (p<0.001).
Gerdes et al., 2016 [21]	Prospective RCT pilot study	Patients with psoriasis receiving online weight loss coaching compared to no intervention	Online coaching (n=8) No coaching (n=9)	Obese patients with psoriasis with stable treatment were enrolled.	No clear change in the severity of psoriasis was noted between groups.

Naldi and colleagues in 2014 conducted a randomized controlled trial (RCT) to assess the relationship between weight reduction and the severity of psoriasis in overweight and obese patients. The study was an RCT with intention to treat and included 303 patients, who were allocated to an intervention arm where they received a 20-week dietary and exercise intervention, and a control group receiving regular diet. The authors found that the intervention group showed significant psoriasis area and severity index (PASI) improvement over the control group. Median PASI reduction in the intervention group was 48% while in the control group it was 25.5% (both 95% CI, p-value 0.02). They concluded that a 20-week dietary and exercise intervention had significantly improved PASI scores in the intervention group compared to controls and thus may be considered as adjunct treatment for obese and overweight patients with psoriasis [19]. Al-Mutairi and Nour in 2014 looked at the effects of weight reduction on the efficacy of biologic treatment in patients with psoriasis. The sample included obese patients with psoriasis on biologic treatment; 262 were randomized to eight weeks of dietary intervention, and the control group received a normal diet. PASI scores were assessed at baseline and then every four weeks until 24 weeks. There was a significant improvement in the PASI scores in the intervention group compared to the control group. At the end of 24 weeks, the diet group lost 12.9 +/- 1.2 kg while the control group only lost 1.5 +/- 0.5 kg. The average PASI score was 85% and 69%, respectively. PASI 75% was achieved by 85.9% of the intervention group compared to 59.3% of the control group (p value <0.001). They concluded that a body weight reduction could improve PASI scores in obese patients on biologics [20].

These findings are consistent with other studies that looked at online coaching as a method for achieving weight loss [21] and with studies that looked at weight loss through surgical intervention such as bariatric surgery [22-25]. Moreover, this review is consistent with results found by the systematic review conducted by Upala and Sanguankeo in 2015 [26] It is worth noting that all studies included in this review demonstrated the clear positive impact weight loss has on the severity of psoriasis symptoms in patients receiving either biologics or oral medication. It is also worth mentioning that studies of longer duration were able to see more significant change in psoriatic severity compared to studies of shorter duration. This is why it is the opinion of this review that studies of short duration do not detect the full extent of clinically  significant changes. Therefore, a study of a duration of more than 12 weeks is going to be necessary to point at the extent of the clinical benefit of weight loss on psoriasis. Finally, is there a difference among diet types (low carbohydrate, ketogenic or vegan/vegetarian diets) on the degree of PASI score improvement? Why does diet impact the severity of symptoms?

It has been hypothesized as an answer to these questions that both psoriasis and obesity are related to an underlying common cause of inflammation. However, this statement, although presented previously in the literature, is not properly delineated and what precisely causes the reduced severity after weight loss is still not clear. Given the added benefit of weight loss over almost all functions of biology and psychology, it is recommended that weight loss becomes a standard step accompanying psoriasis treatment. It will assist in the improvement of symptoms as demonstrated by this review and other published works, and it will also help with the affected self-image that is commonly seen in psoriasis sufferers.

One of the clear limitations of this review is the small sample size of patients receiving weight loss as a treatment compared to the controls. It is also unclear if weight loss in a non-obese patient population can produce similar benefits to patients who are obese, like the samples included in this review. It is the recommendation of this review that further investigation be conducted into what precisely causes the improvement in psoriasis severity after weight loss. Moreover, it has to be investigated if the improvement is seen more in patients receiving low calories or in patients receiving nutritionally dense meals? Finally, a study of the effects of psoriasis in a relatively overweight patient group is important to delineate if this is beneficial to that population.

## Conclusions

The review of the published literature clearly shows that diet and exercise are worthy of consideration as adjunct treatments for psoriasis. Not only do diet and exercise improve the overall health of a patient and are effective in combating oxidative stressors, they also show a positive impact on the PASI scores of patients with psoriasis. It is an easily  accessible, inexpensive, and empowering method to give patients more control over their symptoms. This review recommends that physicians encourage their patients to follow a healthier lifestyle aimed at reducing weight and follow an exercise regimen as a method to improve psoriasis symptoms.
